# Poverty, Weight Status, and Dietary Intake among UK Adolescents

**DOI:** 10.3390/ijerph15061224

**Published:** 2018-06-10

**Authors:** Robert J. Noonan

**Affiliations:** Department of Sport and Physical Activity, Edge Hill University, Ormskirk L39 4QP, UK; Robert.Noonan@edgehill.ac.uk; Tel.: +44-1695-584-488

**Keywords:** adolescent, body mass index, obesity, dietary intake, poverty

## Abstract

The aims of this study were to (1) determine whether an income gradient to overweight and obesity exists in UK adolescents, and (2) examine associations between poverty, weight status, and dietary intake among adolescent girls and boys. Data is from wave six of the UK Millennium Cohort Study. Adolescent height and weight were measured. Body mass index was calculated (kg/m^2^) and used to classify overweight and obesity. Family income and poverty were determined using equivalised household income. Adjusted logistic and multinomial logistic regression analyses were conducted. Ten thousand seven hundred thirty-six adolescents (5425 boys) had complete data. Adolescents in the lowest income group were at greatest risk of overweight and obesity. Adolescents living in poverty were more likely to be overweight and obese, and reported more frequent consumption of sweetened drinks and fast food and less frequent consumption of fruits and vegetables (*p* < 0.001). The magnitude of poverty differences in weight status and dietary intake were greatest among girls. This study evidences a strong income gradient to overweight and obesity among UK adolescents. The findings of this study encourage researchers and policy makers to be equally mindful of the social determinants of health when advocating adolescent behavioural dietary interventions.

## 1. Introduction

Obesity develops from a consistent positive energy balance between intake and expenditure [1]. Roughly one-third of UK children aged between 2–15 years are overweight or obese [2]. Child obesity is related to poor health across the life course [3]. There is strong evidence of an income gradient to child obesity in the UK, such that the burden of obesity falls disproportionately on children from poorer backgrounds [4]. Such inequalities are likely explained by differences in lifestyle behaviours including dietary intake [5,6]. Research in the UK [7,8,9] and other developed countries such as the USA [10] has shown that healthy foods tend to be more expensive than unhealthy foods. Consequently, it is common for poor children to experience a lower quality diet (e.g., high consumption of fast food and low consumption of fruits and vegetables) compared to their more affluent peers [11,12]. Evidence pertaining to adolescents though is limited.

Inequalities in obesity widen during childhood [13,14], but adolescence is considered a period of relative health equality [15]. According to the equalization hypothesis, health inequalities attenuate during adolescence because health is influenced more strongly by peer social relations rather than the social status of the family (which drive child and adult health inequalities) [16]. Equalization has been evidenced in the UK during late adolescence and early adulthood [17] but not during early adolescence.

In addition to family income, gender is considered an important influence on dietary behaviour. For example, several studies have reported higher fruit and vegetable consumption among girls compared to boys [18,19]. However, rarely have studies in this area investigated the effect of poverty on boys’ and girls’ dietary intake separately, or assessed the consumption of healthy and unhealthy food simultaneously. Therefore, the aims of this study were twofold: to (1) determine whether an income gradient to overweight and obesity exists in UK adolescents, and (2) examine associations between poverty, weight status, and dietary intake among adolescent girls and boys.

## 2. Materials and Methods

### 2.1. Participants

Data were derived from wave six of the Millennium Cohort Study (MCS). The MCS is a nationally representative UK sample of children born between September 2000 and January 2002. The first home interviews were conducted with parents and children between 2001 and 2002 when children were aged 9 to 11 months and involved 18,819 children from 18,533 families (for details, see [20]). Five subsequent interviews were conducted with parents and children at age 3, 5, 7, 11, and 14 years. During the age 14 interview, child anthropometric measurements were taken and information on sociodemographic characteristics and family behaviours were collected from parents and children. The present study included participants who had complete ethnicity, family income, weight status, and dietary intake data at age 14 years. The original study received ethical approval from the National Research Ethics Service Research Ethics Committee London-Central (13/LO/1786).

### 2.2. Measures

#### 2.2.1. Child Weight Status

Stature was measured to the nearest millimetre using a portable stadiometer (Leicester Height Measure, Seca, Birmingham, UK), and body mass was measured to the nearest 0.1 kg using Tanita HD-305 scales (Tanita UK Ltd., Middlesex, UK). Body mass index (BMI) was calculated from stature and body mass as a proxy measure of body composition (kg/m^2^). Overweight and obesity were defined using the International Obesity Taskforce (IOTF) age- and gender-specific BMI cut-points [21].

#### 2.2.2. Family Income and Poverty

Parent reported family income was assessed using quintiles of household income equivalised according to the Organisation for Economic Co-operation and Development household equivalence scale. Poverty was defined as having an equivalised household income below 60% of the UK median [22,23].

#### 2.2.3. Child Dietary Intake

Participants reported the frequency with which they consume at least two portions of fruit and vegetables per day. Responses included never, some days, and every day. Responses were collapsed and two dichotomized variables created representing daily fruit and vegetable consumption. Participants reported on a 7-point Likert scale how often they consume sugary drinks (e.g., regular cola or squash), and fast food (e.g., McDonalds, Burger King, KFC). Responses ranged from more than once per day to never. Responses were collapsed and four dichotomized variables were created representing daily and weekly sweetened drink and fast food consumption.

#### 2.2.4. Confounding Factors

Potential confounding factors were selected a priori based on previous evidence [18,24,25]. Participant gender and ethnicity were self-reported. Ethnic group categories were based on census categories [26] and included White, Mixed, Indian, Pakistani and Bangladeshi, Black or Black British, and Other Ethnic group. A dichotomous variable was created to represent White and non-White participants.

### 2.3. Analysis

All analyses were conducted using SPSS v. 24 (SPSS Inc.; Chicago, IL, USA) and statistical significance was set at *p* < 0.05. Descriptive statistics were calculated for all measured variables. Multinomial logistic regression analyses assessed whether an income gradient to overweight and obesity existed among UK adolescents. The lowest income quintile was the reference category. Analyses were adjusted for ethnicity. Logistic regression analyses assessed associations between poverty and each weight status, and dietary intake variable. Exploratory analyses (not shown) revealed differences for boys and girls in the role that poverty had on weight status and dietary intake. Analyses were therefore conducted for the full sample and separately for boys and girls, and were adjusted for gender (full sample) and ethnicity. Variables were entered in a single step for all analyses.

## 3. Results

Data were available for 10,736 participants. The mean participant age was 14.30 years and 50.50% of the participants were male. The ethnic origin of the participants was 79.70% White, which reflects the ethnic demography of the UK population (Table 1; [27]). Almost 72.00% of participants were above poverty level income. The prevalence of overweight and obesity was 26.60% and 7.40%, respectively. Regular fruit and vegetable consumption was low among the sample. The proportion of participants consuming daily fruit and vegetables was 30.90% and 37.60%, respectively. Daily and weekly consumption of sweetened drinks was 23.70% and 68.60%, respectively. Only 1.80% of participants consumed fast food daily and 28.80% reported weekly fast food consumption. Overall, diet quality was better among girls compared to boys. Boys living in poverty consumed more fruit, sweetened drinks, and fast food than girls living in poverty. Similarly, boys not living in poverty consumed more sweetened drinks and fast food compared to girls not living in poverty.

Associations between poverty, weight status, and dietary intake among adolescent girls and boys are presented in Table 2. Boys (Odds ratio; OR = 1.39, 2.04; *p* < 0.001) and girls living in poverty (OR = 1.55, 2.24; *p* < 0.001) were more likely to be classified as overweight and obese compared to boys and girls not living in poverty, respectively. In comparison to adolescents not living in poverty, adolescents living in poverty reported more frequent consumption of sweetened drinks and fast food and less frequent consumption of fruits and vegetables (OR = 1.92–3.61; *p* < 0.001). The magnitude of difference in weight status and dietary intake outcomes between adolescents living and not living in poverty were consistently greater for girls (OR = 1.55–3.62; *p* < 0.001) compared to boys (OR = 1.39–3.60; *p* < 0.001).

Multinomial logistic regression analyses revealed an income gradient to adolescent overweight and obesity (Table 3). The lowest income adolescents were more likely to be overweight and obese compared to the second to least (OR = 1.18, 1.27; *p* < 0.05), third to least (OR = 1.34, 1.81; *p* < 0.001), fourth to least (OR = 1.64, 2.33; *p* < 0.001), and highest income adolescents (OR = 2.10, 4.11; *p* < 0.001).

## 4. Discussion

The aims of this study were to (1) determine whether an income gradient to overweight and obesity exists in UK adolescents, and (2) examine associations between poverty, weight status, and dietary intake among adolescent girls and boys. The study evidences an income gradient to adolescent overweight and obesity in the UK. Poor diet including a high consumption of fast food and sweetened drinks and low consumption of fruit and vegetables was most prevalent among adolescents living in poverty. Disparities in weight status and dietary intake among adolescents living and not living in poverty were greatest among girls.

Extending beyond previous UK research [4], this study evidenced an income gradient to overweight and obesity among UK adolescents. Adolescents in the lowest income quantile were 2.1 and 4.1 times more likely to be overweight and obese compared to adolescents in the highest income quantile, respectively. Previous research has revealed a deprivation and maternal education gradient to child overweight [4]. In this study, poverty was a strong influence on adolescent weight status. These findings challenge the equalization hypothesis and suggest that the family still plays an important role on health at age 14. The present study revealed that poverty differences in weight status were greater among girls compared to boys. These findings are in contrast to results from the English National Child Measurement Programme (NCMP), which showed that deprivation inequalities in child weight status are greater among boys [28]. However, the NCMP results were based on children rather than adolescents who have greater autonomy over their health-related behaviours (e.g., dietary intake and physical activity). Moreover, social disadvantage in the NCMP was assessed using an area-level indicator rather than a more sensitive measure of individual family social-disadvantage, such as household income, which captures more accurately the financial element of social disadvantage. These factors combined may affect the weight status of girls more so than boys.

The study revealed marked poverty differences in diet quality among adolescents. Some previous research in this area has reported small socioeconomic differences in dietary intake quality among adolescents [29]. The research, however, employed a less sensitive measure of social disadvantage (i.e., parental education) and was conducted in Slovakia, which experiences greater income equality than the UK. In the current study, it was revealed that UK adolescents living in poverty were most likely to report a frequent intake of energy dense food including sweetened drinks and fast food alongside limited fruit and vegetable consumption. Food price and family income influence food choices, diet quality, and dietary habits [5]. It is therefore plausible to suggest that the poor dietary intake among adolescents living in poverty in this study is explained in most part by the economic constraints of living in poverty [11]. A constrained family food budget is positively related to a high energy, low nutrient diet [6], and extensive research shows that few disadvantaged adolescents meet fruit and vegetable recommendations [19,30,31]. The findings reported here build on previous research by evidencing the influence of poverty on unhealthy (e.g., sweetened drinks and fast food consumption) and healthy (e.g., fruit and vegetable) food consumption. Moreover, they encourage researchers and policy makers to be equally mindful of the social determinants of health when advocating behavioural interventions to improve diet quality. However, additional longitudinal and experimental research is required to test the cross-sectional associations reported here.

High consumption of sweetened beverages [32] and fast food [33] is positively associated with adolescent obesity, and, as evidenced in the present study, the burden of obesity falls disproportionately on adolescents from poorer backgrounds. Recently, Bann and colleagues evidenced widening socioeconomic inequalities in adolescent obesity in the UK [34]. The present study attempted to explore potential pathways that may explain the link between poverty and adolescent obesity. Obesity, however, is complex and multifactorial, and further research examining the concurrent effect of dietary intake and physical activity on adolescent weight status by poverty is needed. Another novel element of this study was the investigation of dietary intake by poverty among boys and girls separately. Although diet quality was worse among boys compared to girls, the largest poverty differences in dietary intake were observed for girls not boys. Pitel et al. [29] also revealed larger socioeconomic inequalities (assessed from parental education) in nutritional behaviour among girls compared to boys. Together, these findings suggest that adolescent interventions to combat poor diet should consider poverty and gender differences in their design and implementation. Ultimately, intervention programmes to improve adolescent diet quality and health require a complex systems approach through modifications to the physical, social, political, and economic environment in which families make health decisions [35,36].

This study represents the first to examine the influence of poverty on weight status and dietary intake among UK adolescents. The sample was large, heterogeneous, and spanned the whole of the UK. There are also some study limitations to note. The self-reported dietary intake responses may have been subject to measurement error and social desirability bias. Weight status was based on BMI, which reflects both fat and fat-free components of body mass [37] and may underestimate excess body fat mass [38]. Sensitivity analyses were conducted using body fat percentage overweight and obesity classified categories [39]. The results (which are available on request) were largely consistent with those reported using BMI. Weight status inequalities may have been related to other lifestyle factors not examined here, such as physical activity. The study sample was large but the design was cross-sectional and is unable to determine causality.

## 5. Conclusions

At age 14 there was a strong income gradient to overweight and obesity among UK adolescents. Poverty was strongly associated with unhealthy weight status and dietary intake. The magnitude of poverty inequalities in weight status and dietary intake were consistently greater among girls. Overall, the study findings indicate that adolescents living in poverty represent an important target group for future health interventions aimed at improving diet and weight status. There is some evidence that dietary patterns track from adolescence to adulthood [40]. As such, the exposure of poor adolescents to a diet high in energy and low in nutrients is a concern and may have implications for their long-term health. Government policy level interventions are required to reduce poverty inequalities in adolescent diet and obesity to prevent health disparities continuing into adulthood, which convey negative health and economic consequences.

## Figures and Tables

**Table 1:** Descriptive characteristics of sample (*n* = 10,736).

Variable	Boy (*n* = 5425)		Girl (*n* = 5311)	
	Not in poverty (*n* = 3934) %	poverty (*n* = 1491) %	Not in poverty (*n* = 3779) %	poverty (*n* = 1532) %
Weight status				
Overweight	23.50	30.50	25.30	34.20
Obese	5.90	11.50	5.60	11.90
Ethnicity				
White	86.50	61.60	87.90	59.60
Mixed	4.50	5.70	4.30	5.00
Indian	3.20	2.00	2.60	2.20
Pakistani and Bangladeshi	1.50	20.90	1.20	23.50
Black or Black British	2.10	6.40	2.00	5.80
Other Ethnic group	2.10	3.30	2.00	3.90
Family income				
Highest	32.70	0.00	32.60	0.00
Q4	33.10	0.00	32.30	0.00
Q3	28.50	0.00	28.50	0.00
Q2	5.70	42.30	6.60	42.90
Lowest	0.00	57.70	0.00	57.10
Dietary intake				
Fruit ≥ 2 times/day	33.00	22.80	36.00	20.50
Vegetables ≥ 2 times/day	40.80	21.50	46.30	23.70
Sweetened drinks ≥ 1 times/day	21.50	37.00	17.60	31.40
Sweetened drinks ≥ 1 times/week	71.70	81.70	57.20	76.10
Fast food ≥ 1 times/day	1.00	4.20	0.90	3.80
Fast food ≥ 1 times/week	25.00	43.10	21.20	43.20

Note: Overweight and obese according to International Obesity Taskforce (IOTF) classification.

**Table 2:** Logistic regression associations between poverty and weight status and poverty and dietary intake among adolescent girls and boys.

Variable	All (*n* = 10,736) ^1^ OR (95% CI)	Boy (*n* = 5425) ^2^ OR (95% CI)	Girl (*n* = 5311) ^2^ OR (95% CI)
Weight status			
Overweight	1.47 (1.33–1.62) ***	1.39 (1.21–1.60) ***	1.55 (1.36–1.78) ***
Obese	2.14 (1.83–2.49) ***	2.04 (1.64–2.54) ***	2.24 (1.79–2.79) ***
Dietary intake			
Fruit ≥ 2 servings/day	1.92 (1.73–2.13) ***	1.67 (1.44–1.92) ***	2.22 (1.91–2.57) ***
Vegetables ≥ 2 servings/day	2.50 (2.26–2.76) ***	2.39 (2.07–2.76) ***	2.60 (2.26–2.99) ***
Sweetened drinks ≥ 1 times/day	2.25 (2.04–2.49) ***	2.18 (1.90–2.49) ***	2.36 (2.04–2.72) ***
Sweetened drinks ≥ 1 times/week	2.12 (1.91–2.35) ***	1.72 (1.47–2.00) ***	2.53 (2.19–2.91) ***
Fast food ≥ 1 times/day	3.61 (2.65–4.91) ***	3.60 (2.36–5.48) ***	3.62 (2.30–5.70) ***
Fast food ≥ 1 times/week	2.16 (1.97–2.37) ***	1.89 (1.66–2.16) ***	2.50 (2.18–2.86) ***

Note: Overweight and obese according to IOTF classification; ^1^ Adjusted for ethnicity and gender; ^2^ Adjusted for ethnicity; CI: confidence interval; OR: odds ratio; *** *p* < 0.001.

**Table 3:** Multinomial logistic regression associations between family income quantiles and weight status.

Variable	Lowest Income (*n* = 1735) ^1^ OR (95% CI)	Q2 (*n* = 1760) ^1^ OR (95% CI)	Q3 (*n* = 2199) ^1^ OR (95% CI)	Q4 (*n* = 2524) ^1^ OR (95% CI)	Highest Income (*n* = 2518) ^1^ OR (95% CI)
Overweight	Reference	1.18 (1.02–1.36) *	1.34 (1.17–1.55) ***	1.64 (1.42–1.88) ***	2.10 (1.82–2.44) ***
Obese	Reference	1.27 (1.03–1.58) *	1.81 (1.45–2.26) ***	2.33 (1.86–2.93) ***	4.11 (3.14–5.36) ***

Overweight and obese according to IOTF classification; ¹ Adjusted for gender and ethnicity; OR: odds ratio; * *p* < 0.05; *** *p* < 0.001.
